# Impacts of Cognitive Load and Task Initiation Timing on Gait Performance in Parkinson's Disease: A Dual‐Task Paradigm Study

**DOI:** 10.1002/cns.71199

**Published:** 2026-10-03

**Authors:** Shohei Okusa, Takehide Kimura, Kenya Tanamachi, Fuminari Kaneko, Yuka Yamada, Michiyuki Kawakami, Jin Nakahara, Morinobu Seki

**Affiliations:** ^1^ Department of Neurology Keio University School of Medicine Tokyo Japan; ^2^ Parkinson's Disease Center Keio University Hospital Tokyo Japan; ^3^ Department of Physical Therapy, Graduate School of Human Health Sciences Tokyo Metropolitan University Tokyo Japan; ^4^ Department of Rehabilitation Medicine Keio University School of Medicine Tokyo Japan; ^5^ Department of Physical Therapy Tsukuba International University Tsuchiura Japan

**Keywords:** cognitive demand, dual‐task, Parkinson's disease, timing of task onset

## Abstract

**Background:**

Gait impairment in Parkinson's disease (PD), especially under dual‐task conditions, contributes to falls and reduced quality of life. Although the impact of cognitive load has been explored, the effect of the cognitive task's initiation timing relative to walking is unclear. We investigated how variations in cognitive load and the timing of task initiation affect gait performance in PD patients.

**Methods:**

Twelve PD patients and 11 age‐matched healthy controls (HCs) completed single‐task walking and two arithmetic cognitive tasks (serial‐5, serial‐7). In dual‐task trials, the cognitive task either preceded gait initiation (cognitive‐first; C5, C7) or followed it (motor‐first; M5, M7). Gait speed, cycle parameters, and inter‐limb coordination were measured. Cognitive performance was primarily quantified as the answering speed. This exploratory study analyzed the dual‐task data using linear mixed‐effects models (LMM).

**Results:**

In PD patients, gait speed was selectively reduced both by higher cognitive load and by initiating the cognitive task before walking (cognitive‐first), whereas HCs showed no such effects. Inter‐limb coordination of the lower limbs was reduced by higher cognitive load and, in PD patients, by the cognitive‐first condition. The cognitive answering speed differed by task load but not by group.

**Conclusion:**

Gait in PD patients is particularly vulnerable when a high‐load cognitive task is initiated before walking, suggesting that both the cognitive demand and the timing of task onset modulate dual‐task interference. These findings may guide tailored dual‐task assessments and rehabilitation strategies for PD.

## Graphical abstract

In patients with Parkinson's disease, gait was most impaired when a demanding cognitive task was initiated before walking rather than after, with reduced lower‐limb coordination under the same condition. The order of task initiation, not merely dual‐tasking itself, shapes gait vulnerability.

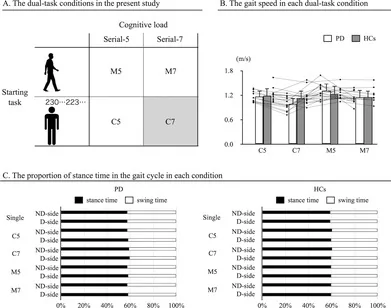

**Abbreviations**
FAB: Frontal Assessment Battery
FOG: freezing of gait
FOG‐Q: Freezing of Gait Questionnaire
HC: healthy control
LEDD: Levodopa Equivalent Daily Dose
MDS: Movement Disorder Society
MDS‐UPDRS: Movement Disorder Society‐Unified Parkinson's Disease Rating Scale
MMSE: Mini‐Mental State Examination
MoCA‐J: the Japanese version of the Montreal Cognitive Assessment
PD: Parkinson's disease
PD‐PIGD: postural instability and gait difficulty subtype of Parkinson's disease

## 1 Introduction

Parkinson's disease (PD), a neurodegenerative disorder characterized by bradykinesia, rigidity, tremor, and gait disturbances, results from the degeneration of dopaminergic neurons in the substantia nigra of the midbrain [1]. Among these symptoms, gait disturbances are particularly common as an initial manifestation among individuals with elderly‐onset PD and are recognized as a major risk factor for falls and fractures, significantly reducing quality of life [2, 3]. Although gait disturbances are typically managed through pharmacological or surgical interventions, the efficacy of these interventions is often limited. The use of non‐pharmacological strategies such as physical therapy and self‐management, particularly for freezing of gait (FOG), has thus become increasingly important [4]. The benefits of these approaches are often modest and transient; however, gait dysfunction remains a major unmet therapeutic need in PD.

In addition to traditional spatiotemporal parameters, recent studies have highlighted the clinical relevance of evaluating the bilateral coordination and step‐to‐step symmetry during gait in patients with PD. These measures capture how well the left and right limbs move in concert and can detect subtle impairments that are not evident from the speed or stride length alone [5, 6]. Impairments in coordination and symmetry have been associated with disease severity and freezing episodes, and pharmacological treatment has been reported to preferentially improve coordination while leaving symmetry relatively unchanged [7]. Such findings suggest that metrics reflecting left–right coordination may provide complementary insights into motor dysfunction and treatment responses beyond what is captured by conventional gait measures.

The postural instability and gait difficulty subtype of PD (PD‐PIGD) is generally less responsive to dopaminergic therapy; it is associated with more pronounced executive and attentional deficits, and it tends to progress more rapidly than the tremor‐dominant subtype [8]. Moreover, individuals with PD‐PIGD are particularly vulnerable in dual‐task situations, in which the simultaneous performance of a motor task and a cognitive task increases the demands on executive and attentional control [9]. Dual‐tasking has been shown to exacerbate gait disturbances in PD, including reductions in gait speed and stride length and increases in gait variability and asymmetry [10, 11, 12, 13]. Functional magnetic resonance imaging studies of PD have demonstrated reduced activity in the brain's supplementary motor area and increased activation in the temporoparietal regions during dual‐tasking, along with decreased cerebellar activity during motor tasks [14]. Based on these findings, dual‐task training has been incorporated into rehabilitation programs to improve functional mobility in PD [15].

The dual‐task paradigm in PD has been explored, and previous studies have manipulated it along several dimensions. The first is the type of the concurrent cognitive task: tasks that load attention and executive function are associated with greater reductions in gait speed, whether drawn from the Trail Making Test and the Frontal Assessment Battery [16] or from working memory, arithmetic, verbal fluency, and Stroop paradigms [17]. The second is graded difficulty within a single cognitive domain, as in the serial‐7 versus serial‐5 contrast, which alters step height in PD [13]. The third is the complexity of the motor task itself, which Meng et al. recently reported to influence dual‐task gait performance more strongly than cognitive complexity, thereby improving the classification of early‐stage PD [18]. Each of these dimensions concerns what is performed concurrently and how demanding it is. The temporal architecture of the paradigm, when each task begins relative to the other, has by contrast been treated as a procedural detail rather than as an experimental variable: in virtually all of these designs, walking is initiated first and the cognitive task is introduced only once a steady‐state gait rhythm has been established. Whether the reverse order, which is arguably at least as common in daily life, alters the magnitude of dual‐task interference in PD has, to our knowledge, never been tested directly.

Building upon the existing evidence that dual‐task interference is modulated by both the cognitive load and attentional allocation, we posited that initiating a cognitively demanding task prior to gait onset would impose greater interference on locomotor control than when the cognitive task is introduced after gait has commenced. Accordingly, we conducted the present study to systematically delineate the combined effects of the cognitive load and the task‐initiation timing on gait performance in individuals with PD, with the broader aim of informing the design of more precise and individualized dual‐task assessment and rehabilitation strategies.

## 2 Methods

### 2.1 Subjects

Twelve patients with PD attending the outpatient clinic at Keio University Hospital and 11 age‐matched healthy controls (HCs) provided their written informed consent to participate in the study between May 2022 and February 2024. The patients' PD diagnoses were based on the Movement Disorder Society (MDS) clinical diagnostic criteria for clinically established or probable PD. The inclusion criteria were as follows: (1) age ≥ 20 years, (2) in a medically stable condition, and (3) with the ability to walk on flat ground without the use of a walking aid. The exclusion criteria were as follows: (1) the presence of another disease that could affect performance in walking or cognitive tasks, and (2) experiencing pain during walking. All 12 of the PD patients were assessed in the ON state, with the timing of medication administration appropriately adjusted. The HCs had no history or signs of a central nervous system disorder and were matched with the PD patients by age and sex.

### 2.2 Survey of the Study Participants' Information

We collected demographic and clinical background information including age, sex, onset age, disease onset timepoint, treatment duration, Hoehn and Yahr stage, Levodopa Equivalent Daily Dose (LEDD), and postural abnormalities such as camptocormia, antecollis, scoliosis, and Pisa syndrome from the participants' medical records and registration information. We assessed the cognitive function of the PD patients and HCs by administering the Mini‐Mental State Examination (MMSE), the Japanese version of the Montreal Cognitive Assessment (MoCA‐J), and the Frontal Assessment Battery (FAB). Motor symptoms were evaluated using the Movement Disorder Society‐Unified Parkinson's Disease Rating Scale (MDS‐UPDRS) Part III, and the severity of gait freezing was assessed with the Freezing of Gait Questionnaire (FOG‐Q).

### 2.3 Experimental Environment

The study was conducted at Keio University Hospital. We prepared an unobstructed, straight, 2‐m‐wide walkway, comprising a 20‐m measured segment with a 2‐m approach segment before the 0‐m mark and a 2‐m walk‐through segment beyond the 20‐m mark. Participants therefore began walking at the start of the approach segment, 2 m before the 0‐m mark, to allow them to establish a stable walking pattern before entering the measured segment. Three infrared sensors (Omron Corporation, Kyoto, Japan) were placed at the 0‐, 2‐, and 20‐m marks of the measured segment. The sensor at 0 m marked entry into the measured segment rather than the onset of walking, and participants continued walking for a further 2 m beyond the 20‐m mark. The sensors at 2 and 20 m were used to calculate the participants' gait speed over the 18 m between them. The infrared sensor at 2 m was also used to trigger the announcement of the starting numbers for the serial‐5 and serial‐7 tasks. This announcement was delivered through a wireless headset (GN Jabra Engage 65 Mono, GN Group, Ballerup, Denmark) worn by the participants.

### 2.4 Task Design

We prepared three types of single‐tasks: (1) a walking motor task, (2) a serial‐5 cognitive task, and (3) a serial‐7 cognitive task. In the walking motor task, the participant was instructed on the length of the walkway at a comfortable pace. Gait speed in the main analyses was calculated from 2 to 20 m. Because the manipulation of dual‐task‐initiation timing would be expected to exert its effect early in the walk, we additionally computed the gait speed from 0 to 2 m as sub‐analysis. For the assessment of gait cycle parameters, we used Trigno Avanti wireless inertial measurement unit sensors (Delsys, Natick, MA, USA). Two sensors were placed on the dorsal side of each of the participant's wrists, and two sensors were placed on the lateral side of each shank (proximal to the lateral malleolus and aligned with the fibula).

We analyzed the gait cycle data by using the angular velocity measurements along the horizontal frontal axis, as described [19]. From the gait cycle analysis, we extracted the average durations of the stride time, stance time, swing time, and the proportion of the stance time in the gait cycle. These analyses were performed separately for the participants' dominant and non‐dominant sides. In the group of PD patients, the side with the most prominent motor symptoms was defined as the dominant side, and in the HCs, the participant's dominant hand was used to determine the dominant side.

For the assessment of inter‐limb coordination during gait, we calculated normalized cross‐correlation values of angular velocities between the right and left upper limbs and between the right and left lower limbs. The maximum value of the normalized cross‐correlation (MCC) was defined as the representative value for each participant (Figure 1). Subsequently, to calculate the amount of change in the MCC (C‐MCC), we subtracted the MCC value obtained under the dual‐task condition from that obtained under the single‐task condition. Thus, a positive C‐MCC value indicates a reduction in inter‐limb coordination during dual‐task walking relative to single‐task walking.

**FIGURE 1:**
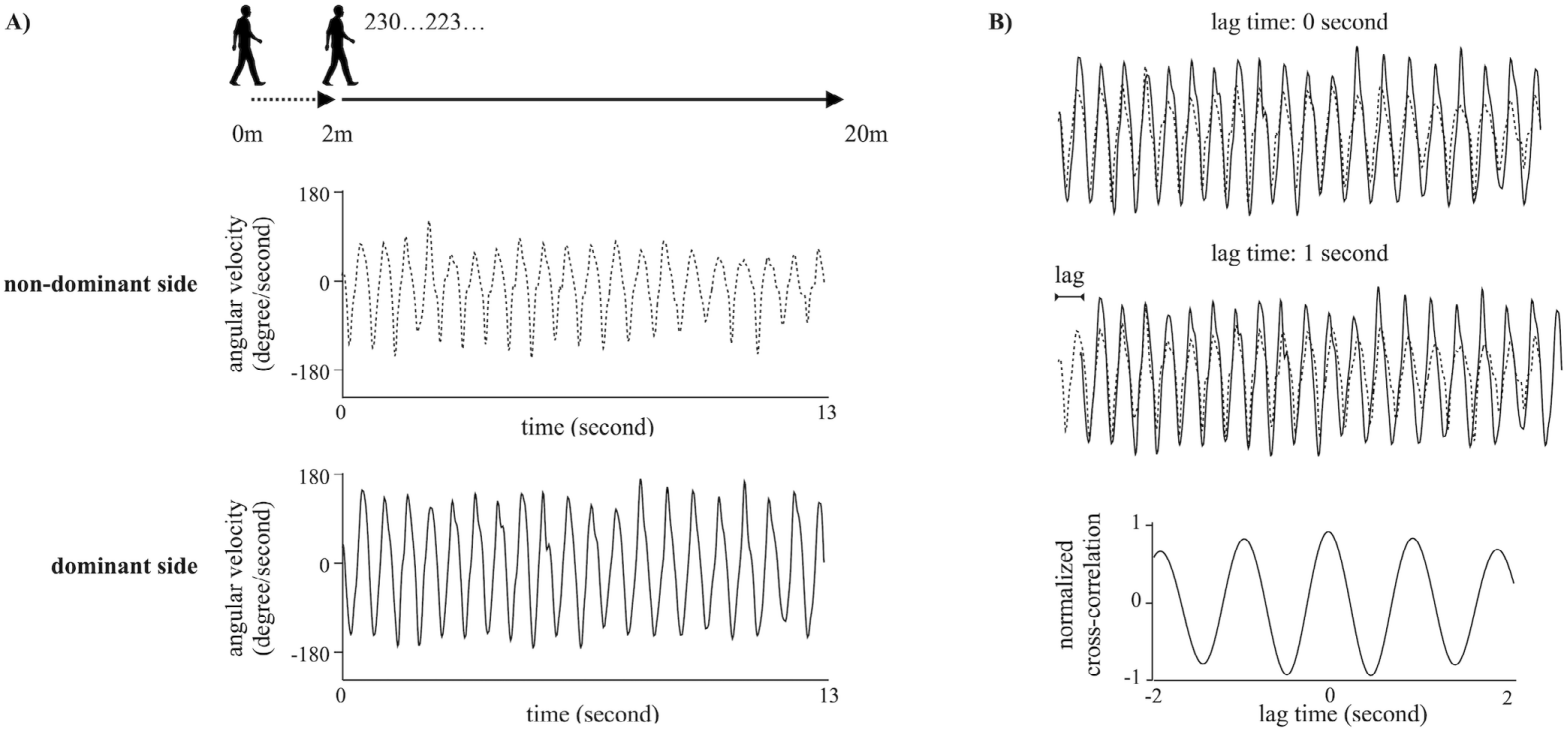
Overview of the calculation of the maximum normalized cross‐correlation (MCC) values. Normalized cross‐correlation values of angular velocities were calculated between limbs for the assessment of the participants' interlimb coordination during gait. (A) For example, we obtained the angular velocities of both wrists. (B) We calculated the correlation coefficient using the obtained angular velocities with a lag time of −2 to 2 s. The maximum value of the normalized MCC was then defined as the representative value for each participant.

For the serial‐5 cognitive task, the participant was seated in a chair and instructed to subtract serial 5 s from a randomly selected three‐digit number for a 30‐s period. For every trial, the three‐digit starting number was drawn at random from the range 200–990 using a pre‐generated list. No starting number was repeated across that participant's single‐task and dual‐task trials. The assignment of starting numbers to conditions was randomized independently for each participant. No systematic difference in arithmetic difficulty, for example, in the number of borrow operations required over the first several subtractions, was introduced between the serial‐5 and serial‐7 conditions or between the cognitive‐first and motor‐first conditions. The starting number was announced via a wireless headset, and the participant responded verbally. His or her responses were recorded on a personal computer via the headset. Responses were judged correct when the participant subtracted 5 accurately from the preceding number. For example, if the starting number was 100 and the sequence of responses was 95, 90, 86, 81, 76…, “86” was judged as an error, but “81” was judged as a correct response. The answering speed (i.e., the number of correct responses per unit time) was used as a main evaluation index for this task. In addition to the answering speed, we computed the following cognitive‐performance indices for each condition. The total responses were defined as the total number of answers the participant produced during the task period. The number of correct responses was the subset of these answers that satisfied the criterion described above, and the accuracy was defined as the number of correct responses divided by the total number of responses. The error rate was defined as one minus the accuracy (i.e., the proportion of incorrect responses among all responses).

The serial‐7 cognitive task followed the same procedure as the serial‐5 cognitive task, except that the participant subtracted serial 7 instead of 5.

By combining the above‐mentioned single‐tasks, we created four dual‐task conditions:

1. C5 dual‐task condition: the serial‐5 cognitive task + the walking motor task (low cognitive load, cognitive‐first; starting with the serial‐5 task)
2. C7 dual‐task condition: the serial‐7 cognitive task + the walking motor task (high cognitive load, cognitive‐first; starting with the serial‐7 task)
3. M5 dual‐task condition: the walking motor task + the serial‐5 cognitive task (low cognitive load, motor‐first; starting with walking)
4. M7 dual‐task condition: the walking motor task + the serial‐7 cognitive task (high cognitive load, motor‐first; starting with walking)

For example, in the C5 dual‐task condition, the participant began by performing the serial‐5 subtraction task while standing. After successfully providing three consecutive answers, the participant was instructed to start walking. The examiner tapped the participant on the shoulder to signal the initiation of walking after the third correct response. In contrast, in the M5 dual‐task condition, the participant began walking in the same manner as in the walking motor task, and upon crossing the infrared sensor placed at 2 m, a three‐digit number was announced through the headset, prompting the participant to perform the serial‐5 subtraction task while continuing to walk.

The single‐ and dual‐tasks were each performed twice. The order in which the tasks were performed was random. Each participant was familiarized with the tasks through practice in advance. The participants were instructed to give equal attention to the dual‐tasks.

### 2.5 Statistical Analyses

The dual‐task cost (DTC) for gait speed and for cognitive answering speed was calculated as: DTC (%) = [(single‐task value − dual‐task value)/single‐task value] × 100, where a positive DTC indicates a reduction in performance under the dual‐task conditions relative to the single‐task conditions.

The present study was an exploratory study. Given the repeated‐measures structure of the data and the relatively small sample, the results were analyzed using linear mixed‐effects models (LMM). For gait speed (2–20 m), gait speed (0–2 m, sub analysis), DTC of gait speed, cognitive answering speed, DTC of cognitive answering speed, accuracy, error rate, total responses, the lower‐limb C‐MCC, and upper‐limb C‐MCC, LMM were fitted with the participant as a random effect (random intercept) and group (PD patients vs. HCs), cognitive load (serial‐5 vs. serial‐7), starting task (cognitive‐first vs. motor‐first), and their interactions as fixed effects.

For the proportion of stance time in the gait cycle, an LMM was fitted with the participant as a random effect and group, dual‐task condition (C5, C7, M5, M7), side (dominant vs. non‐dominant), and their interactions as fixed effects. Models were estimated by restricted maximum likelihood. When a significant interaction was identified, estimated marginal means were compared with Bonferroni correction. An estimated marginal mean difference is reported with 95% confidence intervals (CI). The normality of model residuals was assessed using the Shapiro–Wilk test. All statistical analyses were performed using SPSS ver. 25 (IBM Corp., Armonk, NY).

## 3 Results

### 3.1 Normality and Sensitivity Analysis

Shapiro–Wilk tests indicated that residuals of the lower‐limb C‐MCC model (*p* = 0.090), cognitive answering speed model (*p* = 0.110), and gait speed (0–2 m) model (*p* = 0.477) satisfied the normality assumption, whereas residuals for the remaining models showed statistically significant departures from normality. Detailed results of the Shapiro–Wilk tests, including W statistics, skewness, and kurtosis for all models, are provided in Table S1. Given that LMM are relatively robust to violations of normality and given that the present study was framed as exploratory, all results are reported without data transformation (e.g., logarithmic transformation). Since there were 2 patients with scoliosis, we also investigated the impact on the walking. We therefore excluded these 2 patients and repeated all analyses (*n* = 10 for gait speed and the cognitive outcomes; *n* = 9 for the proportion of stance time and the C‐MCC, reflecting the sensor malfunction described in Section 3.3). The results were consistent with those of the main analysis (Table S2).

A post hoc sensitivity analysis using G*Power (version 3.1) indicated that with 12 PD patients and 11 HCs, the study had 80% power to detect a medium effect size (Cohen's *f* = 0.25) at *α* = 0.05 for the between‐group interaction, based on a repeated‐measures analysis of variance (ANOVA) framework. Although G*Power does not directly support LMM, the repeated‐measures ANOVA framework provides a reasonable approximation of statistical power for the present LMM design given their structural similarity.

### 3.2 Clinical Variables

The characteristics of the PD patients and the HCs are presented in Table 1. The age, sex, and dominant hand were matched between the PD and HC groups. No significant between‐group differences were observed for cognitive function as assessed by the MMSE, MoCA‐J, and FAB.

**TABLE 1:** Clinical characteristics of the patients with Parkinson's disease (PD) and the healthy controls (HCs).

	PD	HCs	*p*‐value
Age, yrs	69.8 ± 8.7	73.3 ± 3.8	0.23
Males: females, *n*	5:7	5:6	0.59
Onset age, years	63.6 ± 9.5	—	—
Disease duration, years	6.3 ± 3.5	—	—
Dominant hand, right/left, *n*	10/2	8/3	0.46
Dominant side of symptoms, right/left, *n*	5/7	—	—
Presence of camptocormia, *n* (%)	7 (58.3)	—	—
Presence of dropped head, *n* (%)	2 (16.7)	—	—
Presence of scoliosis, *n* (%)	6 (50.0)	—	—
Hoehn and Yahr stage	2 (range 2–4)	—	—
LEDD (mg/day)	653.7 ± 391.4	—	—
MMSE score, points	28.9 ± 1.8	29.3 ± 1.1	0.58
MoCA‐J score, points	26.8 ± 2.4	27.6 ± 1.5	0.30
FAB	16.8 ± 1.3	17.5 ± 0.7	0.12
MDS‐UPDRS Part III	28.4 ± 12.1	—	—
FOG‐Q	8.4 ± 6.5	—	—

*Note:* Data are presented as mean ± SD unless otherwise indicated. Hoehn and Yahr stage is presented as median (range). All patients with PD were assessed during the medication ON state.

Abbreviations: FAB, Frontal Assessment Battery; FOG‐Q, Freezing of Gait questionnaire; LEDD, Levodopa Equivalent Daily Dose; MDS‐UPDRS, Movement Disorder Society‐Unified Parkinson's Disease Rating Scale; MMSE, Mini‐Mental State Examination; MoCA‐J, the Japanese version of the Montreal Cognitive Assessment.

### 3.3 Walking Motor Task

Note that angular velocity data could not be obtained for one PD patient due to a sensor malfunction. Consequently, gait speed and cognitive answering speed data were available for all 12 PD patients, whereas the cross‐correlation and gait phase analysis were conducted using data from 11 PD patients.

#### 3.3.1 Gait Speed

As illustrated in Figure 2, the LMM for gait speed (2–20 m) revealed significant main effects of cognitive load (*F*(1, 63) = 26.97, *p* < 0.001, mean difference 0.116, 95% CI [0.071, 0.161]) and starting task (*F*(1, 63) = 14.54, *p* < 0.001, mean difference 0.085, 95% CI [0.041, 0.130]), and significant group × cognitive load (*F*(1, 63) = 7.46, *p* = 0.008) and group × starting task (*F*(1, 63) = 7.26, *p* = 0.009) interactions. The main effect of group and the three‐way interaction were not significant. Decomposition of the group × cognitive load interaction showed that the effect of cognitive load on gait speed was selective to the PD group: in PD patients, gait speed was significantly slower under the serial‐7 condition than under the serial‐5 condition (mean difference 0.177, 95% CI [0.115, 0.239], *p* < 0.001), whereas no such difference was observed in HCs (mean difference 0.055, 95% CI [−0.010, 0.120], *p* = 0.093). Similarly, decomposition of the group × starting task interaction showed that gait speed was significantly slower under the cognitive‐first condition than under the motor‐first condition in PD patients (mean difference 0.145, 95% CI [0.084, 0.207], *p* < 0.001) but not in HCs (mean difference 0.025, 95% CI [−0.040, 0.090], *p* = 0.442).

**FIGURE 2:**
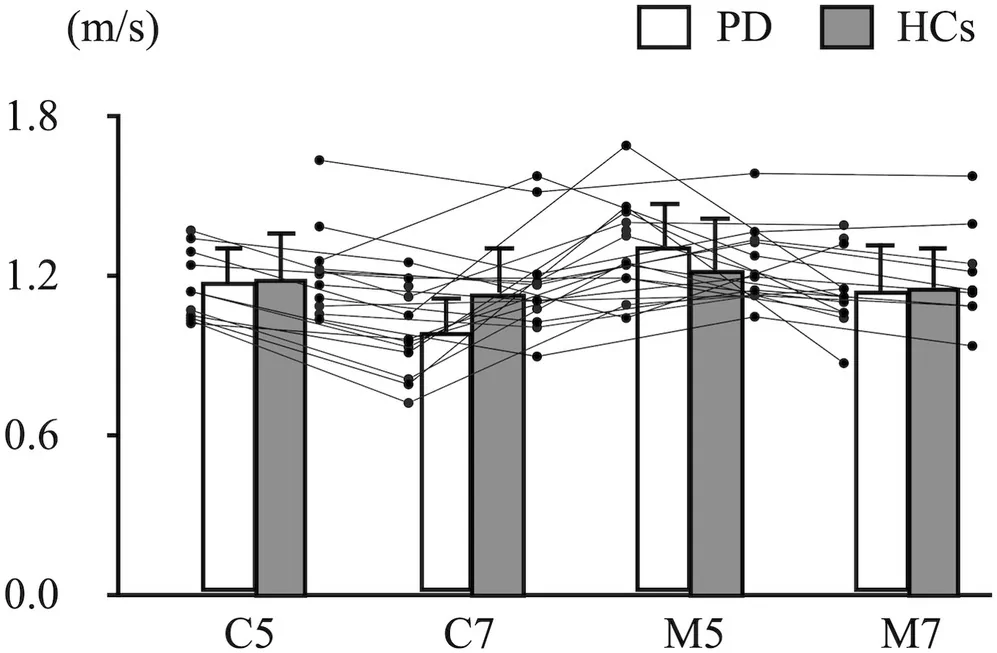
The gait speed results achieved by the patients with Parkinson's disease (PD) and the healthy controls (HCs). Individual data points are connected by lines for each participant. Significant group × cognitive load (*p* = 0.008) and group × starting task (*p* = 0.009) interactions were found. In the PD group, gait speed was significantly slower under serial‐7 than serial‐5 (*p* < 0.001) and under the cognitive‐first than the motor‐first condition (*p* < 0.001), whereas the HCs showed no such differences.

The LMM for gait speed (0–2 m) revealed significant main effects of cognitive load (*F*(1, 63) = 25.01, *p* < 0.001, mean difference 0.097 m/s, 95% CI [0.058, 0.136]) and starting task (*F*(1, 63) = 32.85, *p* < 0.001, mean difference 0.111 m/s, 95% CI [0.072, 0.150]). The other results were non‐significant (Tables S4 and S5).

The LMM for the DTC of gait speed revealed significant main effects of cognitive load (*F*(1, 63) = 7.31, *p* = 0.009, mean difference 4.65, 95% CI [1.21, 8.08]) and starting task (*F*(1, 63) = 42.94, *p* < 0.001, mean difference 11.26, 95% CI [7.83, 14.70]), and a significant group × starting task interaction (*F*(1, 63) = 15.13, *p* < 0.001) and cognitive load × starting task interaction (*F*(1, 63) = 7.31, *p* = 0.009). Decomposition of the group × starting task interaction showed that the DTC of gait speed was significantly larger under the cognitive‐first condition than under the motor‐first condition in PD patients (mean difference 17.95, 95% CI [13.20, 22.70], *p* < 0.001), whereas no significant difference was observed in HCs (mean difference 4.58, 95% CI [−0.39, 9.54], *p* = 0.070). Decomposition of the cognitive load × starting task interaction showed that the DTC of gait speed was significantly larger for serial‐7 than for serial‐5 under the cognitive‐first condition (mean difference 9.29, 95% CI [4.43, 14.15], *p* < 0.001) but not under the motor‐first condition (*p* > 0.999).

#### 3.3.2 The Proportion of Stance Time in the Gait Cycle

The durations of each gait phase are provided in Table 2. The LMM for the proportion of stance time revealed a significant main effect of group (*F*(1, 20) = 5.68, *p* = 0.027), with a lower overall stance proportion in PD patients than in HCs (mean difference 0.016, 95% CI [0.002, 0.030]), a significant main effect of dual‐task condition (*F*(3, 140) = 7.57, *p* < 0.001), and a significant group × side interaction (*F*(1, 140) = 13.25, *p* < 0.001). For the main effect of dual‐task condition, post hoc pairwise comparisons with Bonferroni correction indicated that the stance proportion was significantly higher in the C7 condition than in the C5 (mean difference 0.007, 95% CI [0.000, 0.013], *p* = 0.036), M5 (mean difference 0.011, 95% CI [0.005, 0.017], *p* < 0.001), and M7 conditions (mean difference 0.007, 95% CI [0.001, 0.014], *p* = 0.012), whereas no other pairwise comparison reached significance. Decomposition of this interaction showed that the proportion of stance time differed significantly between the dominant and non‐dominant sides in PD patients (mean difference 0.008, 95% CI [0.004, 0.013], *p* < 0.001), whereas no side difference was observed in HCs (*p* = 0.099) (Figure 3). Furthermore, the between‐group difference in the stance proportion was significant on the non‐dominant side (mean difference 0.022, 95% CI [0.008, 0.036], *p* = 0.004) but not on the dominant side (*p* = 0.162). This indicates that the reduced stance proportion in PD was driven by the non‐dominant side.

**TABLE 2:** The duration of the gait phase in the patients with Parkinson's disease (PD) and healthy controls (HCs).

PD	Single‐task	C5	C7	M5	M7
Stride time, ms					
Dominant	926.7 ± 84.6	946.0 ± 87.1	1009.2 ± 124.3	940.2 ± 74.8	959.7 ± 89.0
Nondominant	926.3 ± 84.4	945.3 ± 87.3	1007.4 ± 124.9	940.3 ± 74.8	958.9 ± 88.9
Stance time, ms					
Dominant	533.0 ± 60.8	551.6 ± 62.6	600.4 ± 92.4	544.1 ± 57.4	559.3 ± 67.6
Nondominant	531.2 ± 66.6	542.5 ± 68.2	592.4 ± 95.7	538.4 ± 62.2	550.7 ± 69.5
Swing time, ms					
Dominant	393.8 ± 26.9	394.5 ± 27.7	408.8 ± 34.5	396.2 ± 21.7	400.3 ± 26.2
Nondominant	395.1 ± 20.8	402.8 ± 20.8	415.0 ± 30.9	401.9 ± 17.7	408.2 ± 23.0

HCs	Single‐task	C5	C7	M5	M7
Stride time, ms					
Dominant	1014.1 ± 72.0	1046.5 ± 87.3	1088.2 ± 101.2	1022.0 ± 77.3	1053.3 ± 101.6
Nondominant	1014.7 ± 72.8	1046.1 ± 86.9	1087.5 ± 100.2	1022.2 ± 77.6	1052.9 ± 101.5
Stance time, ms					
Dominant	598.2 ± 51.4	619.6 ± 58.6	650.8 ± 71.1	605.2 ± 54.0	626.7 ± 71.0
Nondominant	594.3 ± 50.4	631.5 ± 66.7	652.7 ± 76.5	605.0 ± 60.4	630.1 ± 76.6
Swing time, ms					
Dominant	416.0 ± 25.3	426.9 ± 31.1	437.4 ± 32.1	416.8 ± 27.5	426.5 ± 32.8
Nondominant	420.4 ± 29.2	414.6 ± 35.8	434.5 ± 32.1	417.2 ± 20.6	422.8 ± 27.6

*Note:* The data are mean ± SD. C5: walking motor task + serial‐5's cognitive task (starting with the serial‐5's task); C7: walking motor task + serial‐7's cognitive task (starting with the serial‐7's task); M5: walking motor task + serial‐5's cognitive task (starting with walking); M7: walking motor task + serial‐7's cognitive task (starting with walking).

**FIGURE 3:**
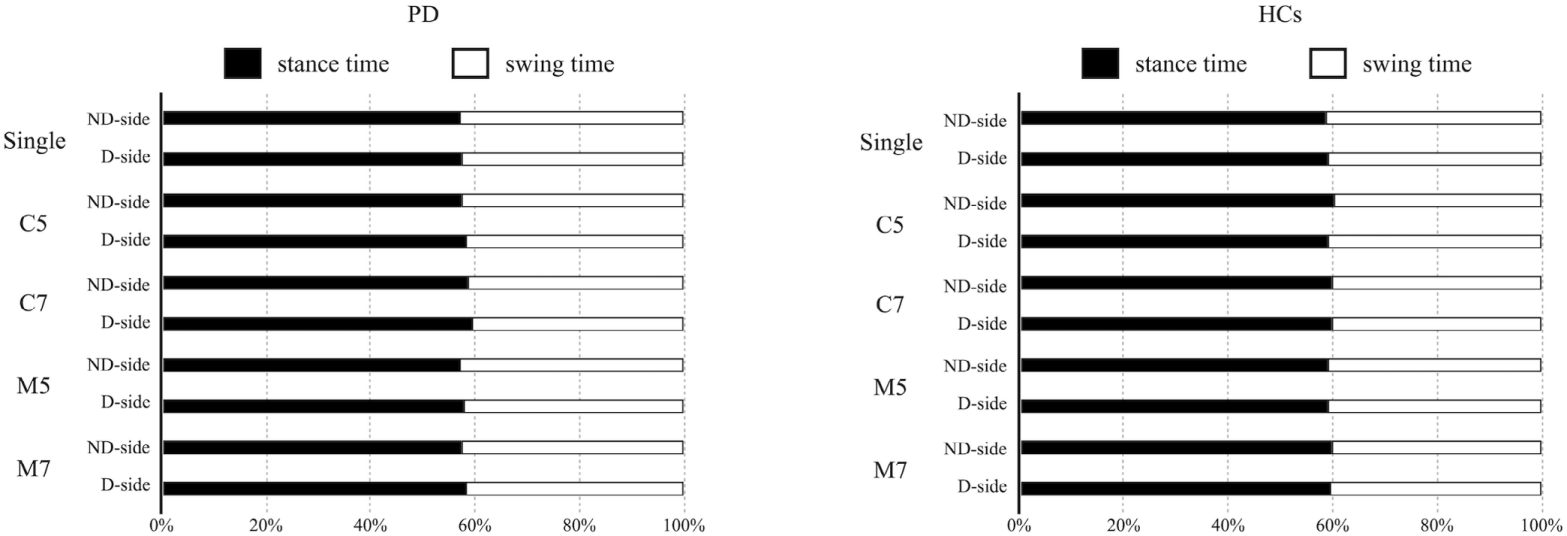
The proportion of stance time in the gait cycle in each condition in the PD and HCs groups. A significant group × side interaction was found (*p* < 0.001): The PD group showed a lower stance proportion on the non‐dominant side than on the dominant side (*p* < 0.001), whereas the HCs showed no side difference.

#### 3.3.3 C‐MCC

The results for the amount of change in the maximum of C‐MCC were shown in Figure 4. For the lower‐limb C‐MCC, the LMM revealed significant main effects of cognitive load (*F*(1, 60) = 20.68, *p* < 0.001) and starting task (*F*(1, 60) = 6.33, *p* = 0.015), and a significant group × starting task interaction (*F*(1, 60) = 4.43, *p* = 0.040); the group × cognitive load interaction approached significance (*p* = 0.059). The lower‐limb C‐MCC was larger under the serial‐7 condition than under the serial‐5 condition (mean difference 0.011, 95% CI [0.006, 0.016], *p* < 0.001). Decomposition of the group × starting task interaction showed that, in PD patients, the lower‐limb C‐MCC was significantly larger under the cognitive‐first condition than under the motor‐first condition (mean difference 0.011, 95% CI [0.004, 0.018], *p* = 0.002), whereas no such difference was observed in HCs (*p* = 0.772).

**FIGURE 4:**
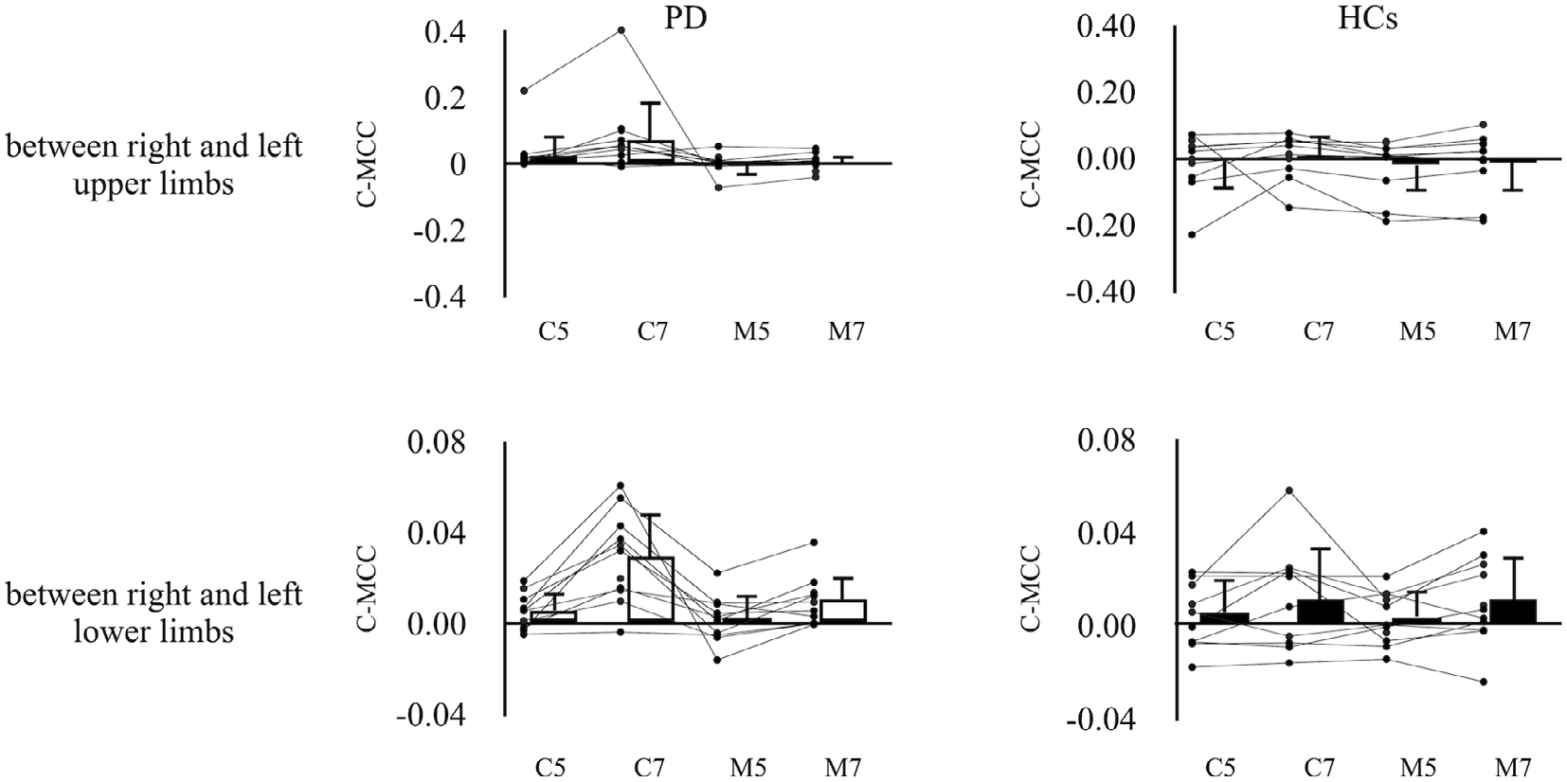
The results for the amount of change in the maximum cross‐correlation (C‐MCC) between right and left limbs (upper panels: Upper limbs; lower panels: Lower limbs). The C‐MCC was calculated by subtracting the MCC under the dual‐task condition from that obtained under single‐task condition. Individual data points are shown for each participant. For the lower limbs, the C‐MCC was larger (i.e., coordination was reduced) under serial‐7 than serial‐5 (*p* < 0.001), and a significant group × starting task interaction (*p* = 0.040) indicated that, in the PD group only, the C‐MCC was larger under the cognitive‐first than the motor‐first condition (*p* = 0.002). For the upper limbs, only a main effect of starting task was found (*p* = 0.011), without a significant group interaction.

For the upper‐limb C‐MCC, the LMM revealed only a significant main effect of starting task (*F*(1, 60) = 6.97, *p* = 0.011; cognitive‐first vs. motor‐first mean difference 0.033, 95% CI [0.008, 0.058]); the group × starting task interaction was not significant (*p* = 0.167). Detailed data for MCC and C‐MCC were shown in Table S3.

### 3.4 The Serial‐5 and Serial‐7 Cognitive Tasks

The results of answering speed, DTC of answering speed, accuracy, error rate, and total responses were shown in Table 3. The LMM for cognitive answering speed revealed a significant main effect of cognitive load (*F*(1, 63) = 287.95, *p* < 0.001), with the serial‐5 condition yielding substantially faster answering speeds than the serial‐7 condition (mean difference 0.646, 95% CI [0.570, 0.722]). No significant main effects of group or starting task were observed, but a significant cognitive load × starting task interaction was found (*F*(1, 63) = 4.52, *p* = 0.038). Decomposition revealed that under the serial‐5 condition, answering speed was significantly faster under the cognitive‐first condition than under the motor‐first condition (mean difference 0.131, 95% CI [0.024, 0.239], *p* = 0.017), whereas no such difference was observed under the serial‐7 condition (*p* = 0.575).

**TABLE 3:** The results of cognitive task in the patients with Parkinson's disease (PD) and healthy controls (HCs).

	Serial‐5	Serial‐7
1. Single‐task condition (performed for a fixed duration of 30 s)		
PD		
Answering speed (response/sec)	0.7 ± 0.2	0.2 ± 0.1
Accuracy (%)	97.8 ± 6.8	76.2 ± 30.4
Error rate (%)	2.2 ± 6.8	23.8 ± 30.4
Total responses	22.4 ± 9.4	7.5 ± 3.9
HCs		
Answering speed (response/s)	0.7 ± 0.1	0.2 ± 0.1
Accuracy (%)	99.7 ± 0.9	83.3 ± 22.3
Error rate (%)	0.3 ± 0.9	16.7 ± 22.3
Total responses	21.5 ± 4.0	7.7 ± 1.9

	C5	C7	M5	M7
2. Dual‐task condition (performed concurrently with walking; duration determined by walking time)				
PD				
Answering speed (response/s)	0.9 ± 0.3	0.2 ± 0.2	0.8 ± 0.3	0.2 ± 0.2
Accuracy (%)	98.8 ± 2.9	62.3 ± 42.2	99.0 ± 2.3	78.3 ± 31.2
Error rate (%)	1.2 ± 2.9	37.7 ± 42.2	1.0 ± 2.3	21.7 ± 31.2
Total responses	15.0 ± 3.8	5.2 ± 3.1	12.1 ± 5.3	4.3 ± 1.9
Dual‐task cost (%)	25.0 ± 24.4	49.7 ± 36.0	43.7 ± 11.4	21.8 ± 75.4
HCs				
Answering speed (response/s)	1.0 ± 0.2	0.2 ± 0.2	0.8 ± 0.2	0.2 ± 0.2
Accuracy (%)	99.6 ± 1.3	68.0 ± 28.9	99.1 ± 2.2	70.0 ± 38.5
Error rate (%)	0.4 ± 1.3	32.0 ± 28.9	0.9 ± 2.2	30.0 ± 38.5
Total responses	16.1 ± 3.9	5.5 ± 2.1	11.1 ± 3.1	4.5 ± 1.3
Dual‐task cost (%)	24.9 ± 11.9	38.0 ± 53.5	48.9 ± 10.3	47.9 ± 39.6

*Note:* The data are mean ± SD. C5: walking motor task + serial‐5's cognitive task (starting with the serial‐5's task); C7: walking motor task + serial‐7's cognitive task (starting with the serial‐7's task); M5: walking motor task + serial‐5's cognitive task (starting with walking); M7: walking motor task + serial‐7's cognitive task (starting with walking).

For accuracy, the LMM revealed a significant main effect of cognitive load (*F*(1, 63) = 19.92, *p* < 0.001), with the serial‐5 condition yielding substantially higher accuracy than the serial‐7 condition (mean difference 33.2, 95% CI [18.3, 48.1]); no significant effects of group, starting task, or any interaction were observed. Error rates mirrored this pattern, with a significant cognitive load main effect (*F*(1, 63) = 42.13, *p* < 0.001, mean difference 29.5, 95% CI [20.4, 38.5]) and no significant group or starting task effects. For total responses, the LMM revealed significant main effects of cognitive load (*F*(1, 63) = 285.43, *p* < 0.001, mean difference 8.71, 95% CI [7.68, 9.74]) and starting task (*F*(1, 63) = 22.34, *p* < 0.001, mean difference 2.44, 95% CI [1.41, 3.47]), and a significant cognitive load × starting task interaction (*F*(1, 63) = 8.96, *p* = 0.004). Decomposition of the cognitive load × starting task interaction for total responses showed that the cognitive‐first condition yielded more responses than the motor‐first condition under the serial‐5 condition (mean difference 3.98, 95% CI [2.52, 5.38], *p* < 0.001). In addition, under the cognitive‐first and motor‐first conditions, total responses were higher under the serial‐5 condition than under the serial‐7 condition (cognitive‐first condition: mean difference 10.26, 95% CI [8.80, 11.71], *p* < 0.001; motor‐first condition: mean difference 7.17, 95% CI [5.71, 8.63], *p* < 0.001). The cognitive DTC of answering speed showed no significant effects of group, cognitive load, starting task, or their interactions.

## 4 Discussion

The present study found that gait speed was significantly reduced in the PD group under the higher cognitive load and under the cognitive‐first starting conditions, whereas no such effects were observed in the HCs group. These group‐specific interactions suggest that PD patients are particularly vulnerable to dual‐task interference when attentional demands are elevated or when the cognitive task is initiated prior to gait.

Although numerous studies have examined dual‐task interference in PD, several focused specifically on the effects of a cognitive task load. As outlined in the Introduction, attentional and executive demand consistently scales dual‐task interference in PD [16, 17], and the serial‐7 versus serial‐5 contrast specifically has been shown to alter gait parameters [13]. These findings are consistent with the interpretation that the serial‐7 task requires greater attention and working memory resources [20], thereby eliciting stronger motor interference, and align with the cognitive load effect observed in the present study. For gait speed over the 0–2‐m segment, significant main effects of cognitive load and task‐initiation order were observed, but neither factor interacted significantly with group. Because the starting number was not announced until participants reached the 2‐m mark, the motor‐first conditions did not involve a concurrent cognitive task during this segment. By contrast, both tasks were underway in both task‐initiation‐order conditions during the 2–20‐m segment. Significant interactions with group were observed for both cognitive load and task‐initiation order only in this later segment, with subsequent decomposition showing that both effects were significant in participants with PD but not in HCs.

The gait speed DTC analyses corroborated these findings from a within‐subject perspective, confirming that the dual‐task cost was selectively amplified in PD patients under the cognitive‐first condition and was greatest when high cognitive load and cognitive‐first initiation coincided (C7). Moreover, the absence of group differences in cognitive answering speed, and the lack of significant effects on the cognitive DTC, indicate that PD patients maintained their cognitive task performance at a level comparable to HCs across all conditions. This pattern of preserved cognitive performance alongside selectively impaired gait suggests that the observed motor costs were not attributable to a speed–accuracy trade‐off in cognitive performance but rather reflect a disease‐specific vulnerability of gait control to dual‐task interference in PD.

Beyond changes in gait speed, our analysis of the proportion of stance time revealed a disease‐specific, side‐dependent pattern. A significant group × side interaction indicated that the PD patients exhibited a lower stance proportion on the non‐dominant side than on the dominant side, whereas the HCs showed no such side difference; the between‐group difference was significant only on the non‐dominant side (Figure 3). This asymmetry is consistent with the well‐recognized lateralized motor presentation of PD and suggests that dual‐task gait control in PD is characterized by a side‐specific reorganization of the stance phase. Because this side effect was independent of the cognitive load and the starting task, it appears to reflect an intrinsic, lateralized feature of PD gait rather than a purely dual‐task–dependent phenomenon. Side‐specific stance asymmetry may therefore constitute an accessible marker of lateralized motor dysfunction.

Previous studies have reported that the stance time tends to increase in PD patients under cognitively demanding conditions, likely reflecting a compensatory strategy to enhance stability during gait [21, 22]. Healthy older adults typically show little or no change in their stance time during similar dual‐task paradigms [23]. However, these earlier investigations focused primarily on overall dual‐task effects and did not examine the laterality of stance‐phase changes. Our findings extend this literature by showing that the redistribution of stance time in PD patients is lateralized, with the non‐dominant side preferentially affected, thereby providing a spatial dimension to the understanding of dual‐task gait control in PD.

The C‐MCC is a kinematic parameter that evaluates inter‐limb coordination during gait. For the lower limbs, our analyses revealed that coordination was reduced under the higher cognitive load condition, and that this effect was selectively amplified in the PD group when the cognitive task was initiated prior to walking, with the most pronounced reduction evident in the C7 condition. For the upper limbs, a main effect of starting task was observed across both groups, without a significant group interaction. This suggests that upper‐limb inter‐limb coordination is modulated by task order regardless of disease status, and the absence of a group‐specific effect indicates that upper‐limb coordination may be less sensitive to the disease‐related attentional deficits that characterize PD. Unlike the studies that used discrete bilateral coordination indices such as the Phase Coordination Index or simple gait asymmetry metrics [5, 6, 7], our use of the C‐MCC revealed condition‐specific reductions in inter‐limb coordination in the lower limbs that were not described in earlier investigations. Notably, the C‐MCC appeared most sensitive under the conditions where both cognitive and motor demands were simultaneously elevated, suggesting that it may detect subtle, context‐dependent motor deficits that are overlooked by conventional measures. This finding has potential clinical implications. Because C‐MCC data can be obtained with the use of wearable inertial sensors and since the C‐MCC reflects real‐time bilateral limb coupling, we propose that the C‐MCC may serve as a practical biomarker to monitor subtle gait impairments during everyday activities and to tailor dual‐task rehabilitation strategies. Identifying patients whose coordination selectively deteriorates under a high cognitive load at gait initiation could inform individualized interventions, for example, cueing strategies or task sequencing, that minimize the risk of falling and optimize the patients' mobility in daily life.

Our study provides novel insights into the role of the task‐initiation timing in dual‐task interference. Task demand itself has been studied, both on the cognitive side [13, 16, 17] and on the motor side [18]. Our cognitive load effect is consistent with this literature. What has not, to our knowledge, been manipulated systematically is when the cognitive task begins relative to gait onset. Our results provide evidence that dual‐task interference in PD is asymmetric with respect to task order. This asymmetry highlights the importance of temporal structuring in dual‐task designs and represents an important avenue for future research. As dual‐task training becomes more prevalent in PD rehabilitation strategies, clarifying this mechanism could contribute to more efficient and personalized intervention protocols.

Cognitive answering speed was faster under the serial‐5 dual‐task conditions than under the single‐task condition. To further explore the mechanism underlying the faster cognitive answering speed observed in the serial‐5 dual‐task conditions, we examined the relationship between step count and response count during task performance. In the C5 condition, the number of steps was approximately equal to the number of responses (HC: 14.3 steps vs. 16.1 responses; PD: 16.5 steps vs. 15.0 responses), suggesting a near 1:1 correspondence between gait cadence and response frequency. This pattern was less pronounced in the M5 condition (HC: 14.0 steps vs. 11.1 responses; PD: 16.0 steps vs. 12.1 responses), in which walking was initiated first and a steady‐state gait rhythm was established before the cognitive task began, potentially reducing the opportunity for entrainment. These observations suggest that the rhythmic structure of walking entrained the temporal pacing of serial‐5 responses, particularly when the cognitive task was initiated prior to gait.

This study has several limitations to consider. First, as an exploratory study, the sample size was relatively small (12 PD patients). This factor limits statistical power and generalizability and increases vulnerability to Type 2 error and to the influence of outliers; the findings therefore require confirmation in adequately powered, multicenter studies. Second, the strength of the statistical evidence varied across the outcomes examined, and our interpretations should be weighted accordingly. For example, the main effects of cognitive load and starting task on gait speed (both *p* < 0.001) and the group × side interaction for the stance proportion (*p* < 0.001) were supported by low *p*‐values with confidence intervals well separated from zero, and we regard these as the most robust findings of the present study. In contrast, several effects reached only conventional significance thresholds (*p* between 0.01 and 0.05), including the group × starting task interaction for the lower‐limb C‐MCC (*p* = 0.040) and the starting‐task main effect for the upper‐limb C‐MCC (*p* = 0.011), the latter of which was not accompanied by a significant group interaction. These findings should therefore be regarded as preliminary and hypothesis‐generating rather than confirmatory. Third, we used the angular velocity rather than acceleration to evaluate the participants' motor function. This may have introduced measurement variability, particularly in the PD patients who presented with tremor. Nevertheless, the key findings of this study appear robust and are likely to represent clinically meaningful results. Fourth, we did not directly assess freezing of gait episodes in this study, and it thus remains unclear whether the effects observed under the C7 condition were associated with increased occurrences of gait freezing. Finally, because participants began walking 2 m before the 0‐m mark, gait initiation occurred outside the measurement window and was not quantified. Thus, whether the two initiation orders differentially affect the initiation phase itself remains untested. Future studies should instrument gait initiation directly.

## 5 Conclusion

In patients with PD, dual‐task interference was not determined simply by the concurrent performance of a cognitive and a motor task, but depended critically on which task was initiated first. Specifically, interference was greater when a high cognitive load was imposed before walking (cognitive‐first) than when walking was established first (motor‐first), and this asymmetry was accompanied by reduced bilateral coordination of the lower limbs. This finding indicates that the order in which the two tasks are initiated is a key determinant of gait vulnerability in PD, a perspective that may be particularly relevant when designing dual‐task training paradigms.

From a clinical perspective, incorporating structured dual‐task paradigms that manipulate both the cognitive load and the timing of task initiation into routine clinical gait assessments could improve the early detection of gait vulnerability, thereby informing individualized interventions and potentially reducing fall‐related hospitalizations and associated healthcare costs.

## Author Contributions

Conceptualization: S.O. and M.S.; methodology, S.O., T.K., K.T., F.K., Y.Y., M.K., J.N., and M.S.; software, T.K., K.T., and F.K.; validation, S.O., T.K., K.T., F.K., and M.S.; formal analysis, S.O., T.K., K.T., and F.K.; investigation, S.O., T.K., K.T., and Y.Y.; resources, S.O., F.K., Y.Y., M.K., J.N., and M.S.; data curation, S.O., T.K., and K.T.; writing – original draft preparation, S.O. and T.K.; writing – review and editing, K.T., F.K., Y.Y., M.K., J.N., and M.S.; visualization, S.O. and T.K.; supervision, M.S.; project administration, M.S.; funding acquisition, M.S.

## Ethics Statement

The study protocol was approved by the Ethics Committee of the Keio University School of Medicine (approval no. 20211045).

## Conflicts of Interest

The authors declare no conflicts of interest.

## Supporting information


**Table S1:** Shapiro–Wilk Normality Tests for LMM Residuals.

**Table S2:** Comparison of the main and sub analysis. The sub analysis conducted the linear mixed‐effects models after excluding two PD patients with scoliosis.

**Table S3:** Raw MCC and C‐MCC change values for upper and lower limbs across all task conditions in the patients with Parkinson's disease (PD) and healthy controls (HCs).

**Table S4:** Gait speed from 0 to 2 m in all task conditions in the patients with Parkinson's disease (PD) and healthy controls (HCs).

**Table S5:** Comparison of the results of the statistical analysis between gait speed from 0 to 2 m and gait speed from 2 to 20 m.

## Data Availability

The data that support the findings of this study are available from the corresponding author upon reasonable request.
