# Supplementary material for: Impacts of Cognitive Load and Task Initiation Timing on Gait Performance in Parkinson's Disease: A Dual‐Task Paradigm Study
Source: CNS Neurosci Ther. 2026 Oct 3;32(10):e71199. doi: 10.1002/cns.71199 (PMC13633552; doi:10.1002/cns.71199)
Supplement: Supplementary file 1 [file CNS-32-e71199-s001.docx]

|  | Single-task | C5 | C7 | M5 | M7 |
| --- | --- | --- | --- | --- | --- |
| PD | 1.34 ± 0.16 | 1.17 ± 0.16 | 0.99 ± 0.19 | 1.25 ± 0.14 | 1.19 ± 0.14 |
| HCs | 1.33 ± 0.23 | 1.18 ± 0.16 | 1.09 ± 0.15 | 1.25 ± 0.18 | 1.19 ± 0.18 |

**Supplementary Table S4.** Gait speed from 0 m to 2 m in all task conditions in the patients with Parkinson's disease (PD) and healthy controls (HCs)

The data are mean ± SD. C5: walking motor task + serial-5's cognitive task (starting with the serial-5's task); C7: walking motor task + serial-7's cognitive task (starting with the serial-7's task); M5: walking motor task + serial-5's cognitive task (starting with walking); M7: walking motor task + serial-7's cognitive task (starting with walking).
