# Supplementary material for: Impacts of Cognitive Load and Task Initiation Timing on Gait Performance in Parkinson's Disease: A Dual‐Task Paradigm Study
Source: CNS Neurosci Ther. 2026 Oct 3;32(10):e71199. doi: 10.1002/cns.71199 (PMC13633552; doi:10.1002/cns.71199)
Supplement: Supplementary file 2 [file CNS-32-e71199-s002.docx]

**Supplementary Table S1.** Shapiro-Wilk Normality Tests for LMM Residuals

| Outcome | W | p | Skewness | Kurtosis |
| --- | --- | --- | --- | --- |
| Gait speed (0-2m) | 0.987 | 0.477 | 0.144 | -0.102 |
| Gait speed (2-20m) | 0.924 | <0.001 | 0.434 | 3.433 |
| DTC of gait speed | 0.963 | 0.011 | -0.227 | 1.437 |
| Proportion of stance time | 0.893 | <0.001 | 1.231 | 9.584 |
| Lower-limb C-MCC | 0.975 | 0.090 | 0.490 | 1.238 |
| Upper-limb C-MCC | 0.806 | <0.001 | 0.651 | 8.311 |
| Cognitive answering speed | 0.977 | 0.110 | 0.431 | 0.922 |
| DTC of cognitive answering speed | 0.797 | <0.001 | -0.723 | 8.554 |
| Accuracy | 0.708 | <0.001 | -2.645 | 9.948 |
| Error rate | 0.890 | <0.001 | 1.169 | 1.660 |
| Total responses | 0.937 | <0.001 | 1.095 | 3.353 |
