# Supplementary material for: Impacts of Cognitive Load and Task Initiation Timing on Gait Performance in Parkinson's Disease: A Dual‐Task Paradigm Study
Source: CNS Neurosci Ther. 2026 Oct 3;32(10):e71199. doi: 10.1002/cns.71199 (PMC13633552; doi:10.1002/cns.71199)
Supplement: Supplementary file 3 [file CNS-32-e71199-s003.docx]

|  | Single-task | C5 | C7 | M5 | M7 |
| --- | --- | --- | --- | --- | --- |
| MCC (upper limbs) |  |  |  |  |  |
| PD | 0.89 ± 0.07 | 0.86 ± 0.12 | 0.82 ± 0.17 | 0.90 ± 0.06 | 0.89 ± 0.05 |
| HCs | 0.82 ± 0.11 | 0.83 ± 0.13 | 0.81 ± 0.11 | 0.84 ± 0.10 | 0.83 ± 0.11 |
| MCC (lower limbs) |  |  |  |  |  |
| PD | 0.97 ± 0.01 | 0.96 ± 0.01 | 0.94 ± 0.03 | 0.96 ± 0.01 | 0.96 ± 0.01 |
| HCs | 0.96 ± 0.02 | 0.95 ± 0.01 | 0.95 ± 0.02 | 0.96 ± 0.01 | 0.95 ± 0.01 |
|  |  |  |  |  |  |
|  | Single-task | C5 | C7 | M5 | M7 |
| C-MCC (upper limbs) |  |  |  |  |  |
| PD | - | 0.03 ± 0.06 | 0.07 ± 0.11 | 0.00 ± 0.03 | 0.00 ± 0.02 |
| HCs | - | 0.00 ± 0.09 | 0.01 ± 0.07 | -0.02 ± 0.08 | -0.01 ± 0.09 |
| C-MCC (lower limbs) |  |  |  |  |  |
| PD | - | 0.01 ± 0.01 | 0.03 ± 0.02 | 0.00 ± 0.01 | 0.01 ± 0.01 |
| HCs | - | 0.01 ± 0.01 | 0.01 ± 0.02 | 0.00 ± 0.01 | 0.01 ± 0.02 |

**Supplementary Table S3.** Raw MCC and C-MCC change values for upper and lower limbs across all task conditions in the patients with Parkinson's disease (PD) and healthy controls (HCs)

The data are mean ± SD. MCC: maximum cross-correlation; C-MCC: change in MCC (C-MCC = MCC single-task − MCC dual-task); C5: walking motor task + serial-5's cognitive task (starting with the serial-5's task); C7: walking motor task + serial-7's cognitive task (starting with the serial-7's task); M5: walking motor task + serial-5's cognitive task (starting with walking); M7: walking motor task + serial-7's cognitive task (starting with walking).
