# Supplementary material for: Impacts of Cognitive Load and Task Initiation Timing on Gait Performance in Parkinson's Disease: A Dual‐Task Paradigm Study
Source: CNS Neurosci Ther. 2026 Oct 3;32(10):e71199. doi: 10.1002/cns.71199 (PMC13633552; doi:10.1002/cns.71199)
Supplement: Supplementary file 4 [file CNS-32-e71199-s004.docx]

**Supplementary Table S5.** Comparison of the results of the statistical analysis between gait speed from 0 m to 2 m and gait speed from 2 m to 20 m

| Effect | 0–2 m | 2–20 m |
| --- | --- | --- |
| Group | F(1,21) = 0.26, p=.613 | F(1,21) = 0.38, p=.546 |
| Cognitive load | F(1,63) = 25.01, p<.001 | F(1,63) = 26.97, p<.001 |
| Starting task | F(1,63) = 32.85, p<.001 | F(1,63) = 14.54, p<.001 |
| Group × cognitive load | F(1,63) =0.99, p=.325 | F(1,63) = 7.46, p=.008 |
| Group × starting task | F(1,63) = 1.91, p=.172 | F(1,63) = 7.26, p=.009 |
| Cognitive load × starting task | F(1,63) = 3.76, p=.057 | F(1,63) = 0.01, p=.921 |
| Group × cognitive load × starting task | F(1,63) = 1.16, p=.285 | F(1,63) = 0.16, p=.687 |
