# Supplementary material for: Impacts of Cognitive Load and Task Initiation Timing on Gait Performance in Parkinson's Disease: A Dual‐Task Paradigm Study
Source: CNS Neurosci Ther. 2026 Oct 3;32(10):e71199. doi: 10.1002/cns.71199 (PMC13633552; doi:10.1002/cns.71199)
Supplement: Supplementary file 5 [file CNS-32-e71199-s005.docx]

**Supplementary Table S2.** Comparison of the main and sub analysis. The sub analysis conducted the linear mixed-effects models after excluding two PD patients with scoliosis.

|  | Main analysis | | Sub analysis | |
| --- | --- | --- | --- | --- |
|  | F value | p value | F value | p value |
| 1. Gait speed (m/s) |  |  |  |  |
| Cognitive load | 26.97 | < .001 | 20.63 | < .001 |
| Starting task | 14.54 | < .001 | 11.89 | .001 |
| Group × Cognitive load | 7.46 | .008 | 4.36 | .041 |
| Group × Starting task | 7.26 | .009 | 5.44 | .023 |
| 2. Gait speed DTC (%) |  |  |  |  |
| Cognitive load | 7.31 | .009 | 6.52 | .013 |
| Starting task | 42.94 | < .001 | 31.67 | < .001 |
| Group × Starting task | 15.13 | < .001 | 9.33 | .003 |
| Cognitive load × Starting task | 7.31 | .009 | 6.52 | .013 |
| 3. Proportion of stance time |  |  |  |  |
| Group | 5.68 | .027 | 6.58 | .019 |
| Cognitive load | 8.47 | .004 | 6.16 | .014 |
| Group × Side | 12.10 | < .001 | 12.71 | < .001 |
| 4. C-MCC lower limbs |  |  |  |  |
| Cognitive load | 20.68 | < .001 | 15.06 | < .001 |
| Starting task | 6.33 | .015 | 5.47 | .023 |
| Group × Starting task | 4.43 | .040 | 3.81 | .056 |
| 5. C-MCC upper limbs |  |  |  |  |
| Starting task | 6.97 | .011 | 6.43 | .014 |
| 6. Cognitive answering speed (responses/s) |  |  |  |  |
| Cognitive load | 287.95 | < .001 | 314.75 | < .001 |
| Cognitive load × Starting task | 4.52 | .038 | 7.89 | .007 |
| 7. Cognitive speed DTC (%) |  |  |  |  |
| Starting task | 42.94 | < .001 | 31.67 | < .001 |
| Group × Starting task | 15.13 | < .001 | 9.33 | .003 |
| Cognitive load × Starting task | 7.31 | .009 | 6.52 | .013 |

Only significant effects (p < .05) and effects with borderline significance are shown. Non-significant effects not listed were also non-significant in the sub analysis.
